# Estimation of Potentially Toxic Elements Contamination in Anthropogenic Soils on a Brown Coal Mining Dumpsite by Reflectance Spectroscopy: A Case Study

**DOI:** 10.1371/journal.pone.0117457

**Published:** 2015-02-18

**Authors:** Asa Gholizadeh, Luboš Borůvka, Radim Vašát, Mohammadmehdi Saberioon, Aleš Klement, Josef Kratina, Václav Tejnecký, Ondřej Drábek

**Affiliations:** 1 Department of Soil Science and Soil Protection, Faculty of Agrobiology, Food and Natural Resources, Czech University of Life Science, Kamýcká 129, 165 21- Suchdol, Praha 6- Prague, Czech Republic; 2 Laboratory of Image and Signal Processing, Institute of Complex Systems, Faculty of Fisheries and Protection of Waters, University of South Bohemia in České Budějovice, Zámek 136 37 333- Nové Hrady, Czech Republic; Dowling College, UNITED STATES

## Abstract

In order to monitor Potentially Toxic Elements (PTEs) in anthropogenic soils on brown coal mining dumpsites, a large number of samples and cumbersome, time-consuming laboratory measurements are required. Due to its rapidity, convenience and accuracy, reflectance spectroscopy within the Visible-Near Infrared (Vis-NIR) region has been used to predict soil constituents. This study evaluated the suitability of Vis-NIR (350–2500 nm) reflectance spectroscopy for predicting PTEs concentration, using samples collected on large brown coal mining dumpsites in the Czech Republic. Partial Least Square Regression (PLSR) and Support Vector Machine Regression (SVMR) with cross-validation were used to relate PTEs data to the reflectance spectral data by applying different preprocessing strategies. According to the criteria of minimal Root Mean Square Error of Prediction of Cross Validation (RMSEP_cv_) and maximal coefficient of determination (R^2^_cv_) and Residual Prediction Deviation (RPD), the SVMR models with the first derivative pretreatment provided the most accurate prediction for As (R^2^_cv_) = 0.89, RMSEP_cv_ = 1.89, RPD = 2.63). Less accurate, but acceptable prediction for screening purposes for Cd and Cu (0.66 ˂ R^2^_cv_) ˂ 0.81, RMSEP_cv_ = 0.0.8 and 4.08 respectively, 2.0 ˂ RPD ˂ 2.5) were obtained. The PLSR model for predicting Mn (R^2^_cv_) = 0.44, RMSEP_cv_ = 116.43, RPD = 1.45) presented an inadequate model. Overall, SVMR models for the Vis-NIR spectra could be used indirectly for an accurate assessment of PTEs’ concentrations.

## Introduction

Our society and civilization now rely heavily on the mining industry to sustain our way of living. However, mining is one of the anthropogenic activities that causes some of the most dramatic disturbances to the earth. Fertile, cultivated land is transformed into wasteland, as mining activities generate a vast amount of solid wastes, which are deposited at the surface and typically occupy a huge area [1, 2]. Among the various geo-environmental impacts of mining, contamination of soil is by far the most significant. Elevated concentrations of Potential Toxic Elements (PTEs) in soils do not only impact the soil quality, but due to their persistent nature and long biological half-lives, can accumulate in the food chain and can eventually influence human health [3–5]. Although the adverse effects of PTEs have long been known, and exposure to PTEs continues (and is even increasing in some areas), most of the former metallurgical tailing dumpsites are now abandoned. However, no particular safety measures are in place, and their environmental impact has received little attention. Potentially Toxic Elements (PTEs) concentrations in soils can be measured, but their determination depends on large-scale sampling and physical or conventional analysis techniques, which are time-consuming, inefficient, and expensive when applied on a large scale [6]. Moreover, according to Xie et al. [5], conventional methods for environmental soil monitoring require the collection of numerous samples, followed by laboratory analyses that involve complex processes such as separation and pre-concentration. In practice, sampling density and analytical diversity are frequently less than sufficient due to the significant costs of analyses.

Diffuse reflectance spectroscopy technique is a low cost technique with little or no sample preparation, and has been considered as an alternative to conventional soil analytical methods [7, 8]. It has shown to be a powerful tool for such studies in agricultural applications as it provides knowledge of the state of soil, giving results on-site in real-time. Furthermore, this method can be adjusted to provide results for more than one soil attribute of the soil with a single analysis [9]. Some researchers used Visible (Vis) and Near Infrared (NIR), ranging from 350 nm to 2500 nm, to analyze the spectrally active properties of sediment and soil samples. Toxic elements in soils can often be absorbed or bound by these spectrally active constituents [10]. This makes it possible to study the characteristics of PTEs in soils using Vis and NIR spectroscopy [3]. Kemper and Sommer [11] successfully used reflectance spectroscopy to estimate arsenic (As), iron (Fe), mercury (Hg), lead (Pb), sulfur (S) and antimony (Sb) contents in the Aznalcollar Mine area in Spain. Bray et al. [12] also used Vis, NIR and Mid-Infrared (MIR) spectroscopy, calibrated by ordinal logistic regression, for the screening of either contaminated or uncontaminated soil at different thresholds for copper (Cu), zinc (Zn), cadmium (Cd) and Pb. It is believed that Vis-NIR can substantially decrease both the time and costs associated with screening for PTEs.

Chemometric methods are often needed to analyze the spectra characteristics of soil [13]. Using a set of well-known calibration methods makes this process feasible. Choosing the most robust calibration technique can help to achieve a more reliable prediction model. Multiple Linear Regression (MLR) [14], Principle Component Regression (PCR) [15], or Partial Least Squares Regression (PLSR) [10, 16] have been used in the past to build models for estimating the content of toxic elements in soil or sediments. All of the above-mentioned calibration methods require the creation of robust and generalized models, due to their potential tendency to over-fit the data [8, 17]. Therefore, using a method that can overcome the problems of other calibration methods, such as Support Vector Machine Regression (SVMR) seems essential. According to Vapnik [17], SVMR is a supervised non-parametric statistical learning technique; thus it represents a different model class compared with the previous techniques.

To the best of our knowledge, the SVMR technique has not yet been used to analyse soil contamination, in the spectral domain. This study was conducted to assess selected PTEs, namely manganese (Mn), Cu, Cd, Zn, Fe, Pb and As concentrations in anthropogenic soils on brown coal mining dumpsites using Vis-NIR. We evaluate the feasibility of the technique for the rapid prediction of the above-mentioned contaminants, and to compare the performance of PLSR and SVMR methods for multivariate calibrations using soil reflectance spectra. It was envisaged that this rapid and inexpensive method for obtaining accurate information of PTEs would be valuable in providing reference data for soil environment monitoring by proximal and remote sensing.

## Materials and Methods

### 2.1. Study Area and Soil Sampling

**Fig 1:**
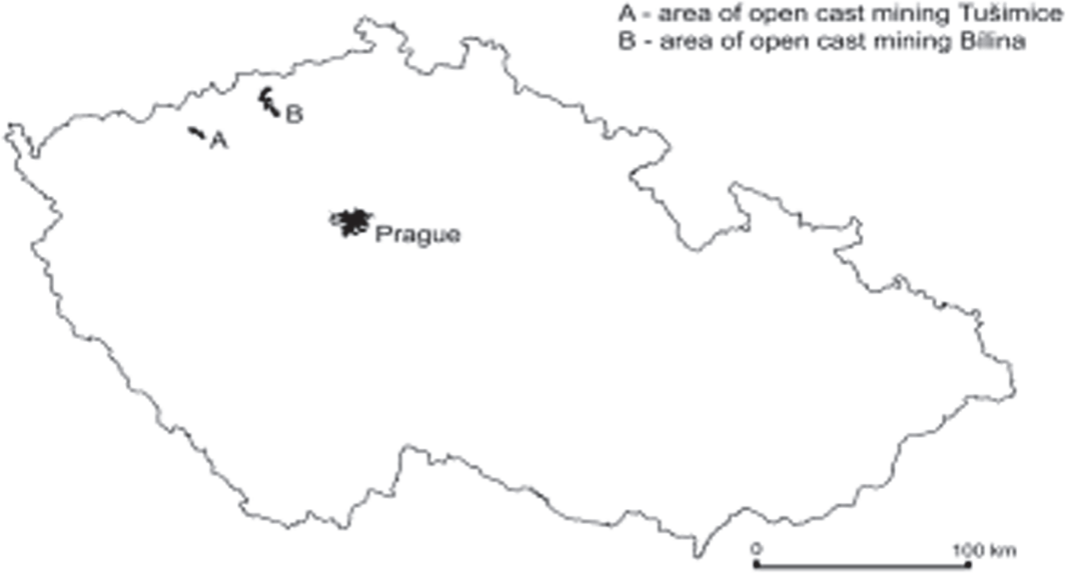
Map of the sampling locations in the Czech Republic.

Six dumpsites, which were located in mines Bílina and Tušimice (Fig. 1), in the Czech Republic, were selected: Pokrok (50° 60’ N; 13° 71’ E), Radovesice (50° 54’ N; 13° 83’ E), Březno (50° 39’ N; 13° 36’ E), Merkur (50° 41’ N; 13° 30’ E), Prunéřov (50° 42’ N; 13° 28’ E) and Tumerity (50° 37’ N; 13° 31’ E). Permission to enter the studied areas was issued by the mining company Severoceske Doly, a.s., Chomutov (www.sdas.cz), which manages these areas, and soil sampling was made under the supervision of its representatives. The land is protected until the reclamation process is completed, for security reasons rather than for natural protection. No protected species were collected.

All dumpsites are formed by clay. On a part of each dumpsite, a cover with natural topsoil was spread in an amount of approximately 2500 to 3000 t per ha one year before sampling. Topsoil material originated from humic horizons of natural soils of the region, particularly Vertisols, and partly also Chernozems (clayic and haplic). Topsoil was not mixed with the dumpsite material. Individual soil properties differed slightly between the six dumpsites. Some characteristics of the soils, including pH, Soil Organic Matter (SOM) and texture were measured using bulk control subsamples since they are important environmental indicators. Specifically, the soil pH range for the area was 5.3–8.5. The SOM content range was 0.6–3.8%. Texture analysis, which was performed by the hydrometer method, showed that soil of the area had 37.30% clay, 33.10% sand and 29.60% silt. Disturbed and undisturbed soil samples were randomly collected at all of the dumpsites randomly. 103 soil samples were collected on the Pokrok dumpsite, 40 samples on the Radovesice dumpsite, 25 samples on the Březno, 38 samples on the Merkur dumpsite, 48 samples on the Prunéřov dumpsite, and 10 samples on the Tumerity dumpsite. Approximately half of the sampling points were located on the area with natural topsoil cover, and on the area without the cover. Sampling was made in the depth of 0 to 20 cm. This depth corresponds to the common depth of a ploughing soil layer, as these soils will be used as arable land in future. Where it was applied, the depth of the topsoil cover was also at least 20 cm.

### 2.2. Soil Analysis

Samples were air-dried, then sieved through a 2 mm mesh. All samples were then used for analyses of PTEs (including Cu, Mn, Cd, Zn, Fe, Pb and As) and reflectance measurements. Target elements were extracted using 2M HNO_3_ [5, 18]. Arsenic was determined in extracts by a flow-through electrochemical coulometry analyser EcaFlow 150GLP (Istran, SK). All other elements were measured by atomic absorption spectrometry (Varian Spectra AA280, Varian, Australia). Samples and standards were matrix matched and all analyses were performed in triplicates.

### 2.3. Reflectance Spectroscopy Measurement

Reflectance was measured in the 350–2500 nm wavelength range by a FieldSpec 3 spectroradiometer (Analytical Spectral Devices Inc., USA) with a contact probe under standard laboratory conditions. The spectral resolution of the spectroradiometer was 3 nm for the region 350–1000 nm and 10 nm for the region 1000–2500 nm. Furthermore, the radiometer bandwidth from 350–1000 nm was 1.4 nm, and from 1000–2500 nm, bandwidth was 2 nm. Samples were illuminated using a stable direct current powered 50 W tungsten-quartz-halogen lamp, which was mounted on a tripod. The angle of incident illumination was 15^°^ and the distance between the illumination source and the sample was 30 cm. A fiber-optic probe with 8^°^ field of view was used to collect reflected light from the sample. The probe was mounted on a tripod and positioned approximately about 10 cm vertically above the sample. The sample dish was over-filled with soil sample, and then leveled off using a blade to ensure a flat surface that is flush with the top of the dish. The final spectrum was an average based on 20 iterations from 4 directions, with 5 iterations per direction to increase the signal-to-noise ratio. Each sample spectrum was corrected for background absorption by division of the reference spectrum of a standardized white BaSO_4_ panel.

### 2.4. Model Construction and Validation

Outliers are commonly defined as observations that are not consistent with the majority of the data [19], such as observations that deviate significantly from normal values. An outlier can be defined as (i) a spectral outlier when the sample is spectrally different than the rest of the samples, and (ii) a concentration outlier, which occurs when the predicted value has a residual difference significantly greater than the mean of the predicted values (>2.5 times). For all samples, an exploratory analysis was carried out to detect outliers before establishing the regression model [3]. Murray [20] noted that removing outliers may increase prediction accuracy; hence the outliers were left out. Correlation between PTEs concentration and reflectance spectra was determined using Pearson’s correlation.

It was necessary to calibrate a model that provides accurate predictive performance about the quantity of PTEs contained in each soil sample; the captured soil spectra, together with laboratory data of PTEs were imported into R software (R Development Core Team, 2011) to be processed. From a total of 264 samples, subsets were mostly used to determine the content of PTEs. The number of samples subjected to individual analysis was then as follows: the entire data were tested for Mn and Fe; 148 samples were tested for Pb; 115 for Cu and Zn, and 104 samples for Cd and As. Spectral preprocessing techniques are a variety of mathematical methods for correcting light scattering in reflectance measurements and data enhancement before the data was used in calibration models. Spectral derivative transformation is actually one of the best methods for removing baseline effects [21]. The first derivative is very effective for removing baseline offset; the second derivative is very effective for both the baseline offset and linear trend from a spectrum [21, 22]. In this study, prior to all further spectra treatments, the noisy part of the spectra range (350–399 nm and 2450–2500 nm) was cut out and then the spectra were subjected to Savitzky-Golay smoothing with a second-order polynomial fit and 11 smoothing points [6, 10]. This was done in order to remove the artificial noise caused by the spectroradiometer instrument. Predictive models were fitted based on smoothed raw spectra at first, and then two types of preprocessed spectra were used, as with first and second derivative manipulations which were calculated using the Savitzky-Golay algorithm. Moreover, PLSR and SVMR models were employed to calibrate spectral data with chemical reference data, and to describe the relationship between reflectance spectra and measured PTEs.

The PLSR technique is closely related to Principle Component Regression (PCR). However, unlike PCR, the PLSR algorithm selects successive orthogonal factors that increase the covariance between predictor (X, spectra) and response variables (Y, laboratory data). By fitting a PLSR model, one hopes to find a few PLSR factors that explain most of the variation in both predictors and responses [23]. Partial Least Squares Regression (PLSR) handles multicollinearity. It is robust in terms of data noise and missing values, and unlike PCR, it balances the two objectives of explanation response and predictor variation (thus calibrations and predictions are more robust) and presents the decomposition and regression in a single step [23]. PLSR models were fitted with the *pls* R package [24], using the classical orthogonal scores algorithm. The optimal number of latent variables (factors) was determined by minimizing the value of Root Mean Square Error of Prediction (RMSEP) by leave-one-out cross validation [5].

The concept of SVMR follows a different approach of supervised learning. Its algorithm is based on the statistical learning theory [25]. It has been known to strike the right balance between accuracy attained on a given finite amount of training patterns, and an ability to generalize to unseen data. The most valuable properties of SVMs are their ability to handle large input spaces efficiently, to deal with noisy patterns and multi-modal class distributions, and their restriction on only a subset of training data in order to fit a non-linear function [8, 26]. For SVMR prediction we used radial basis function kernel, as contained in *e1071* R package [27].

Because a limited number of samples were available, the validation was done using the leave-one-out cross validation procedure with computing corresponding index of determination in cross validation (R^2^_cv_) and Root Mean Squared Error of Prediction of Cross Validation (RMSEP_cv_). The leave-one-out cross validation means that in turn, one sample was taken out from the data set and the calibration was made based on the remaining samples, and then the prediction was made for the sample that was taken out at the beginning. Each time, *n-1* samples were used to build the regression model from all *n* samples within the dataset [19]. The same procedure is repeated for each sample in the sample set. The differences between observed and predicted values give the R^2^_cv_ and RMSEP_cv_, which is a standard method for validation of the prediction models [5, 6].

### 2.5. Accuracy Assessment of Techniques

Assessment of prediction accuracy of the models was carried out using a leave-one-out cross-validation approach (R^2^_cv_ and RMSEP_cv_) [5, 28, 29, 30], the value of RPD was also calculated which is an useful indicator of the practical utility of a calibration model to predict soil property considering the variation of the soil property [5]. The RMSEP_cv_ and RPD were computed as follows:

$$\mathrm{RMSE}P_{\mathrm{cv}}=\sqrt{\frac{1}{N}\sum_{i=1}^{N}{({y^{\prime }}_{i}-y_{i})}^{2}}$$

where y´_i_ is the predicted and y_i_ is the observed value. The smallest RMSEP_cv_ value was related to the optimal calibration model.

$$\mathrm{RPD}=\frac{\mathrm{SD}}{\mathrm{RMSE}P_{\mathrm{cv}}}$$

where SD is Standard Deviation.

## Results and Discussion

### 3.1. Soil Samples Descriptive Statistics

General statistical results of PTEs in the six dumpsites are summarized in Table 1.

**Table 1:** Descriptive statistics of PTEs in the studied sample set according to location.

Item	Cu	Mn	Fe	Cd	Pb	Zn	As
	mg/kg						
*Pokrok (n = 103)*							
Min	5.50	198.3	2503.4	0.01	7.60	8.30	0.49
Max	35.70	869.1	9752.6	0.73	42.40	127.10	19
Mean	13.76	599.4	5418	0.27	18.43	25.26	4.48
Std.	3.58	118.6	1330.1	0.11	5.32	15.77	3.39
C.V. (%)	26	20	25	40	29	62	76
*Radovesice (n = 40)*							
Min	6.42	254.1	1754.4	0.03	4.70	9.38	0.18
Max	22.10	844.1	6876.9	0.30	49.60	66.85	1.30
Mean	14.20	541.3	4489.3	0.17	13.70	21.98	0.67
Std.	3.45	125.1	974.4	0.05	6.40	11.15	0.25
C.V. (%)	24	23	22	30	47	51	38
*Březno (n = 25)*							
Min	9.01	473.3	2398.5	0.00	10.90	11.49	0.49
Max	38.81	885.8	31281.8	0.37	21.60	200.27	5.89
Mean	14.37	680.9	9967.2	0.16	14.17	41.50	1.12
Std.	5.95	105.9	103.58.2	0.11	2.97	41.62	1.04
C.V. (%)	41	16	104	64	21	100	93
*Merkur (n = 38)*							
Min	7.29	318	2361.8	0.04	9.30	6.95	0.33
Max	16.76	787.3	8047.7	0.27	55.90	32.22	9.57
Mean	12.22	590	4852.7	0.16	17.53	13.56	0.97
Std.	1.77	100.7	1355.6	0.06	7.23	4.19	1.45
C.V. (%)	14	17	28	39	41	31	149
*Prunéřov (n = 48)*							
Min	8.40	41.6	2105	0.00	0.90	6.60	0.00
Max	92.24	984	9225.4	0.24	24.80	213.11	3.30
Mean	15.81	552.6	5532.5	0.11	14.38	26.83	0.98
Std.	14.36	224.4	1595.5	0.06	4.82	39.32	0.86
C.V. (%)	91	41	29	55	34	147	87
*Tumerity (n = 10)*							
Min	12.29	496.8	4163.8	0.00	9.50	15.50	0.37
Max	20.34	1027.6	8484.3	0.20	14.50	48.56	0.51
Mean	15.03	753.1	6702.3	0.12	12.25	25.61	0.42
Std.	2.40	192.3	1426.6	0.05	1.38	10.32	0.05
C.V. (%)	16	26	21	44	11	40	12

A comparison of Coefficients of Variation (C.V.) of different contaminants showed that among all parameters, As had the highest C.V., especially in the Merkur area (149%). Hence As varied the most as compared to other measured parameters. In contrast, Pb in Tumerity (11%) had the lowest C.V., which shows that its distribution is more homogenous than the other PTEs.

The estimated mean concentrations of Cd (0.27 mg/kg), Pb (18.43 mg/kg) and As (4.48 mg/kg) were higher in Pokrok than other locations, which may suggest a threat toward soil destined for agricultural soils. The mean concentration of Fe (9967.19 mg/kg) in the studied soil sample of Březno was also high, which was probably related to the iron oxide-rich characteristic of the soil type and its formation.

### 3.2. Vis-NIR Reflectance Spectroscopy of the Soil Samples and Data Preprocessing

**Fig 2:**
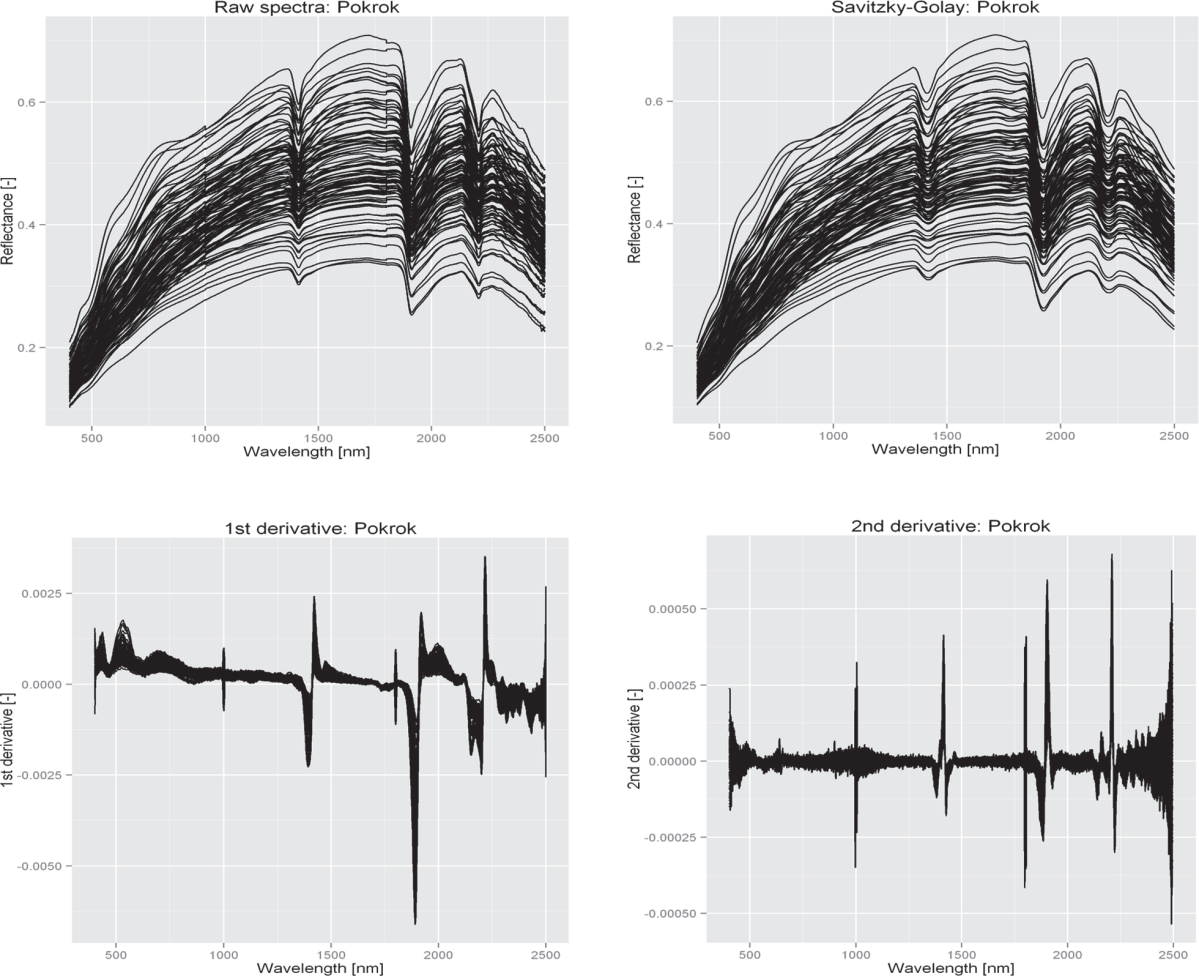
Raw reflectance spectra, smoothed spectra by Savizky-Golay and preprocessed spectra of soil samples for Pokrok.

Visual inspection of the spectra allowed detection of some spectral readings, possibly affected by measurement errors. These were removed, and the final spectral library had a total of 264 soil spectra. Raw reflectance, smoothed spectra by Savitzky-Golay, and first and second derivative spectra of all selected soil samples in Pokrok (the location that had the most samples) are shown in Fig. 2. Other locations also showed the same pattern.

As can be seen in Fig. 2 (raw spectra), sets of spectra were characterised qualitatively by observing the positive and negative peaks, which occur at specific wavelengths [23]. Positive peaks are due to the component of interest, while negative peaks correspond to interfering components [31]. Due to the presence of the same spectrally active properties in all locations, the Vis-NIR spectra of all soil sample sets were similar. The characteristic wavebands of reflectance spectra were only around 1400, 1900, and 2200 nm. However, there were more features of high variability at around 460–550, 1400, 1900–2000 and 2200 nm in the first derivative. The second derivative showed a similar spectrum in all locations. Stenberg et al. [32] indicated that the first and second derivatives were by far the most popular spectral preprocessing techniques for soil property prediction using Vis and NIR spectroscopy.

From Fig. 2, it is clear that three essential absorption bands are evident throughout all the compressed spectra (around 1400, 1900 and 2200 nm). Also, the general shape and slopes of all the curves are similar. The regions around 1400 and 1900 nm were related to vibrational frequencies of OH groups in the water and hydroxyl absorption, and the features around 2000–2500 nm were related to the characteristics of soil organic matter and clay minerals [6, 33]. According to Song et al. [10], although intense bands in the Vis-NIR spectra are not directly associated to the presence of PTEs or other constituents of interest in this paper, it is clear that PTEs can interact with the main spectrally active components of soil. Based on this phenomenon, chemometric models can be developed for soil samples in order to screen their toxic element concentrations. Similar results were reported by Ben-Dor et al. [34], Janik et al. [35], Kooistra et al. [36, 37], Wu et al. [3], Ren et al. [6] and Song et al. [10].

### 3.3. Matrix Correlation of PTEs and Reflectance Spectra

**Fig 3:**
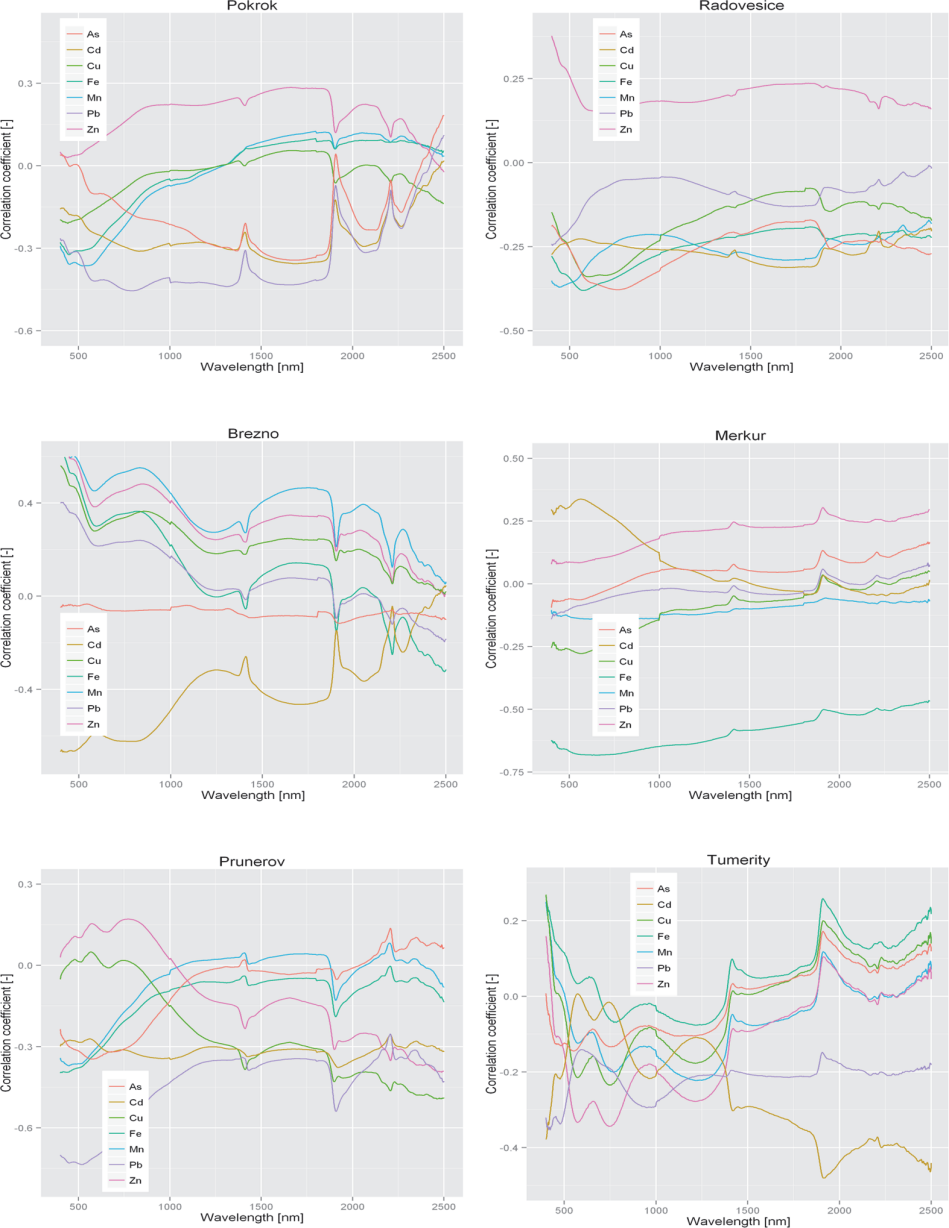
Correlation between reflectance of Vis-NIR and PTEs in different locations.

An easy approach to visualize spectral implications of PTEs is to plot correlation spectra (i.e. the correlation between the attributes and measured reflectance for each wavelength). As was shown by Song et al. [10], linear correlation coefficients between reflectance and PTEs were low to moderate. For example, in Březno was observed-0.6 < r < 0.6 throughout the Vis and NIR regions (Fig. 3). However, this indicates that PTEs contribute to the reflectance of almost all wavelengths. Fig. 3 also shows that the concentrations of toxic elements in six dumpsite soil samples displayed complex changes in their correlations with the Vis-NIR reflectance of soil spectra. Moreover, it can be seen that each element exhibits its maximum correlation coefficient at a different wavelength. Correlation analysis also indicates that the correlation coefficients of Cd and Pb, are usually separated from the other elements. Wu et al. [38] obtained the same results; they related this to week correlation of these elements (Cd, Pb) with spectra and Fe.

At each location, the PTEs were categorized into two or three groups according to their behavior and relationships with soil Vis-NIR spectra. This grouping is useful to more easily recognize the prediction ability of Vis-NIR spectroscopy for PTEs with similar behavior. Moreover, it simplifies the estimation of prediction accuracy of each PTE in a group. In Pokrok, PTEs were categorized into two groups. The first group of elements (Cd, Pb and As) had stronger negative correlation coefficients with spectral bands than the second group (Cu, Zn, Fe and Mn); the first group displayed relatively moderate negative spectral correlation at 786 nm for Pb, and the second group had the highest correlation at 1667 nm for Zn (Fig. 3). However, correlation coefficient changes of PTEs with Vis-NIR spectra of the Prunéřov dumpsite, in contrast to dumpsite Pokrok categorized into three groups, namely (Mn, As and Fe), (Zn and Cu) and (Cd and Pb), but it also displayed the strongest correlations for Pb (at 513 nm) and for Zn (at 769 nm).

In Radovesice, the highest positive, and also the highest negative, spectral correlations can both be seen in the first group of elements (Zn, Cu, As and Fe), in which the strongest positive correlation coefficient related to Zn at 401 nm. Fe represented the lowest spectral correlation at 578 nm arising from Fe^3+^ absorption. These results were similar to results of Ben-Dor [39]. They mentioned that the contribution of the region 390–550 nm is attributed to the spectral absorption features of free iron oxides. The Březno dumpsite also exhibited the highest positive and negative correlation coefficients in the Vis region, at 401 nm (Zn) and 433 nm (Cd), respectively. Correlation coefficient changes of all PTEs at the Merkur dumpsite exhibited similar behavior to Vis-NIR spectra, and categorized into one group. Similar to results of Vohland et al. [40], the order of correlation coefficients between the PTEs in this dumpsite was Cd > Zn > As > Pb > Mn > Cu > Fe, and the highest positive and negative correlation coefficients again belonged to the Vis region (561 nm to 651 nm, respectively).

Clearly, correlation changes in the Vis region of the first group of PTEs (Fe, As, Cu and Zn) in the Tumerity dumpsite fell into the 410–540 nm range. Correlation changes of Cd, which categorized into the second group of PTEs (Cd and Pb); fell into the NIR region with the highest negative correlation coefficient at 1913 nm (around the water absorption band). Stenberg et al. [32] also reported the same results.

An earlier report by Song et al. [10] indicated similar results for agricultural soils of the Changjiang River Delta, China. These findings provide support for the use of diffuse reflectance spectra in predicting the PTEs contents of soil samples.

Active soil constituents such as SOM and soil texture, as well as the indirect relationship between chromophores and PTEs can affect the reflectance of PTEs and their correlation with whole reflectance spectra [5, 10, 34]. For example, Kooistra et al. [36] found that there was a positive correlation between the SOM content and the contents of Zn and Cd in floodplains along the river Rhine in the Netherlands, and that increasing SOM content, reflectance of Zn and Cd was changed. Song et al. [10] also mentioned that the wavelength bands with highest correlation for Pb and Cd should correspond to SOM, which suggested that associations with SOM may be the main form of Pb and Cd binding in soils. Their results also showed that Cr, Cu and As had stronger negative correlation coefficients with the spectral bands attributable to the absorption features of clay and organic matter, suggesting that they are strongly bound to these soil constitutes [10].

### 3.4. Multivariate Analysis Using PLSR and SVMR and Validation Test

The first derivative technique was selected as the most suitable preprocessing technique [21]. Multivariate calibration techniques such as PLSR and SVMR have been used to extract soil PTEs calibration models from the reflectance spectra of soils in the Vis and NIR. The adequacy of each calibration model was evaluated based on the value of R^2^_cv_, RMSEP_cv_ and the RPD [41].

As can be seen in Table 2, the two modeling strategies considered in this study provide different prediction accuracies of the studied PTEs. For the PLSR calibration set, R^2^_cv_ values ranged between 0.44 and 0.61. Good and excellent R^2^_cv_ (R^2^_cv_ > 0.81 and R^2^_cv_ > 0.90, respectively) [28] were not obtained for any of the studied elements. The best predictive models were obtained for As (R^2^_cv_ = 0.61, RMSEP_cv_ = 2.98, RPD = 1.81), followed by Cd (R^2^_cv_ = 0.57, RMSEP_cv_ = 0.11, RPD = 1.68). Inadequate models (high RMSEP_cv_ and low R^2^_cv_ and RPD) were obtained for Mn, Zn and Fe. The large variability of the sample set (colour and texture of samples from different dumpsites) used in this study also affects the accuracy of PLSR calibration models developed for PTEs.

**Table 2:** Statistical results for calibration and cross-validation of Vis-NIR diffuse reflectance spectroscopy for each PTEs.

		PLSR			SVMR		
PTE	*n*	R^2^_cv_	RMSEP_cv_	RPD	R^2^_cv_	RMSEP_cv_	RPD
Cu	115	0.50	6.28	1.45	0.78	4.08	2.29
Mn	264	0.44	116.43	1.45	0.58	101.25	1.75
Fe	264	0.48	1619.03	1.32	0.71	1141.08	2.04
Cd	104	0.57	0.11	1.68	0.78	0.08	2.31
Pb	148	0.51	3.12	1.50	0.66	2.24	1.97
Zn	115	0.45	21.84	1.42	0.71	14.51	2.16
As	104	0.61	2.98	1.81	0.89	1.89	2.63

Kooistra et al. [37] predicted Cd and Zn contents in the floodplains of the river Rhine in the Netherlands using high-resolution reflectance spectra based on laboratory measurements with 69 soil samples. They reported very good and satisfactory predictions for both Cd (R^2^_cv_ = 0.94, RMSEP_cv_ = 0.68) and Zn (R^2^_cv_ = 0.95, RMSEP_cv_ = 80.974). Wu et al. [3] reported approximate and acceptable predictions for Cu (R^2^_cv_ = 0.79, RMSEP_cv_ = 6.01), Pb (R^2^_cv_ = 0.81, RMSEP_cv_ = 5.3), Zn (R^2^_cv_ = 0.79, RMSEP_cv_ = 12.83) and also As (R^2^_cv_ = 0.65, RMSEP_cv_ = 1.23) in soils of the Nanjing area, China. In the study of Xie et al. [5], the models provided fairly accurate predictions for Fe (R^2^_cv_ > 0.80, RMSEP_cv_ = 2.74, RPD > 2.00), less accurate, but acceptable for screening purposes for Cu, Pb, and Cd (0.50 < R^2^_cv_ < 0.80, RMSEP_cv_ = 44.05, 4.66 and 0.41 respectively, 1.40 < RPD < 2.00) and poor accuracy for Zn (R^2^_cv_ < 0.50, RMSEP_cv_ = 5.84, RPD < 1.40). Due to this variability, researchers tended to develop calibration models for each field that they measured with Vis and NIR spectroscopy [42, 43]. Moreover, Dunn et al. [44] indicated that the poor predictive ability of Vis and NIR for many soil constituents might result from a poorly distributed sample set with a small range, rather than the inability of Vis and NIR to predict the soil property. Besides sample variation, sample distribution and sample size are all critical to a successful Vis-NIR calibration [5].

To the best of our knowledge, the SVMR technique has not yet been commonly used to analyse and predict PTEs in the spectra domain. In the current work, SVMR was also used to develop prediction models. The results of the SVMR model for Cu, Mn, Fe, Cd, Pb, Zn and As in Vis-NIR spectra are shown in Table 2. Among the studied PTEs, As is the most accurately predicted with SVMR (R^2^_cv_ = 0.89, RMSEP_cv_ = 1.89, RPD = 2.63). This prediction accuracy is classified to be very good. The calibration results for Mn were not as good as the results of the other elements (R^2^_cv_ = 0.58, RMSEP_cv_ = 101.25, RPD = 1.75), indicating a fair model. Results obtained with SVMR for Cu, Fe, Cd, Pb and Zn gives a good classification, although the prediction accuracy of Fe and Pb is slightly lower than those of Cu and Cd.

Table 2 indicates that in the validation procedure, cross-validation R^2^ (R^2^_cv_) of PLSR ranged between 0.44 for Mn and 0.61 for As, while the range for SVMR was between 0.58 for Mn to 0.89 again for As. Based on R^2^_cv_ and RMESP_cv_, which has been introduced as standard methods for validation of the prediction models, in both calibration and validation, the best estimates were clearly obtained for As prediction. Generally, R^2^_cv_ and RMESP_cv_ and also RPD for both methods were satisfactory, but the same as for calibration, SVMR results were more reliable which emphasizes the need for using more flexible techniques, such as SVMR.

By comparing the results of the PLSR and SVMR models for the Vis-NIR spectra, it can be seen that the use of PLSR has been generally successful to calibrate many soil variables including some PTEs concentrations [5, 10, 45]. In this study, PLSR showed moderate predictions, however; SVMR provided fairly good correlations between soil spectra and various PTEs; better prediction was achieved using SVMR, which outperformed the PLSR. From a practical point of view, the prediction accuracies obtained with these two methods generally seem to be acceptable for a number of agricultural applications, including soil science research. The superior performance of SVMR over PLSR can be explained by the inclusion of nonlinear and interaction effects, as well as linear combinations of variables. It is able to approximate nonlinear functions between multidimensional spaces [7, 46, 47].

## Conclusion

This study demonstrated the application of laboratory Vis-NIR reflectance spectroscopy for the prediction of PTEs, including Cu, Mn, Cd, Zn, Fe, Pb and As, using soil samples taken from six brown coal mining dumpsites of the Czech Republic. For each parameter, Vis-NIR calibration models were created by PLSR and SVMR algorithms. Correlation analysis revealed that PTEs contribute to the reflectance of almost all wavelengths, and that the patterns of correlation coefficients of Cd and Pb are usually separated from the other elements. The results showed obvious differences in predictability and accuracy of PLSR and SVMR. When using a SVMR model, soil spectroscopy was shown to be a very promising method for the determination of PTE concentrations in anthropogenic soils. The best predictability of Vis-NIR reflectance spectroscopy was obtained by SVMR for As (R^2^ > 0.90, RMSEP_cv_ = 1.89, RPD > 2.5), followed by Cd, Cu, Zn, Pb and Mn. In generally, our results confirmed that Vis-NIR reflectance spectroscopy combined with first derivative and SVMR methods have great potential for site-specific soil monitoring in high-risk regions. It leads to overoptimistic performance in the assessment of PTEs, which generally involves conducting large numbers of analyses in a short time. For future investigations, hyperspectral sensors may be useful, and have to be explored for fitting specific spectral regions and for models to optimize the estimation of PTEs content.
